# Dual-Mode Bio-CM ^2^ : Multimodal Computational Miniature Mesoscope for Fluorescence and Label-Free Imaging

**DOI:** 10.64898/2026.09.08.750281

**Published:** 2026-09-14

**Authors:** Qilin Deng, Guorong Hu, Bradley C. Rauscher, Marcelo Villafuerte, Bethany Weinberg, Zhixiong Chen, Hui Feng, Anna Devor, Martin Thunemann, Lei Tian

## Abstract

Understanding biological function often requires complementary information on molecular identity, morphology, behavior, and physiology acquired simultaneously from the same specimen. Achieving this capability in miniature microscopes remains challenging because integrating multiple imaging contrasts typically increases optical complexity while compromising field of view (FOV), spatial resolution, or form factor. Here we present dual-mode Bio-CM ^2^ , a multimodal computational miniature mesoscope that simultaneously captures fluorescence and label-free reflectance through a shared optical architecture based on distributed computational optics. The system enables frame-interleaved, co-registered dual-mode imaging over a *∼* 7.5 *×* 10 mm ^2^ FOV with *∼* 6 *µ* m lateral resolution. We demonstrate the versatility of dual-mode Bio-CM ^2^ across diverse biological systems. In freely moving *Caenorhabditis elegans* , reflectance captures body posture and locomotor behavior that contextualize fluorescently labeled protein aggregates. In freely swimming larval zebrafish, reflectance captures whole-body morphology and swimming behavior, while fluorescence visualizes cardiac activity. In head-fixed mice, simultaneous fluorescence and reflectance imaging across the bilateral dorsal cortex provides complementary measurements of neuronal calcium activity and intrinsic hemodynamic signals. By integrating molecularly specific fluorescence with complementary reflectance contrast, dual-mode Bio-CM ^2^ extends distributed computational optics into a scalable platform for multimodal miniature imaging.

## Full Text Availability

The license terms selected by the author(s) for this preprint version do not permit archiving in PMC. The full text is available from the preprint server.

